# Clinical and biological markers predictive of treatment response associated with metastatic pancreatic adenocarcinoma

**DOI:** 10.1038/s41416-023-02170-9

**Published:** 2023-02-22

**Authors:** Alimu Dayimu, Lorena Di Lisio, Shubha Anand, Isart Roca-Carreras, Wendi Qian, Abdulrahman Al-Mohammad, Bristi Basu, Juan W. Valle, Duncan Jodrell, Nikos Demiris, Pippa Corrie

**Affiliations:** 1 Clinical Trials Unit, Department of Oncology, University of Cambridge, Cambridge, UK; 2 Cancer Molecular Diagnostics Laboratory, Department of Oncology, University of Cambridge, Cambridge, UK; 3 Cambridge Clinical Trials Unit, Cambridge University Hospitals NHS Foundation Trust, Cambridge, UK; 4 Oncology Department, Cambridge University Hospitals NHS Foundation Trust, Cambridge, UK; 5 Department of Oncology, University of Cambridge, Cambridge, UK; 6 University of Manchester and The Christie NHS Foundation Trust, Manchester, UK; 7 Department of Statistics, Athens University of Economics and Business, Athens, Greece

**Keywords:** Tumour biomarkers, Predictive markers

## Abstract

**Background:**

Chemotherapy for metastatic pancreatic adenocarcinoma (PDAC) offers limited benefits, but survival outcomes vary. Reliable predictive response biomarkers to guide patient management are lacking.

**Methods:**

Patient performance status, tumour burden (determined by the presence or absence of liver metastases), plasma protein biomarkers (CA19-9, albumin, C-reactive protein and neutrophils) and circulating tumour DNA (ctDNA) were assessed in 146 patients with metastatic PDAC prior to starting either concomitant or sequential nab-paclitaxel + gemcitabine chemotherapy in the SIEGE randomised prospective clinical trial, as well as during the first 8 weeks of treatment. Correlations were made with objective response, death within 1 year and overall survival (OS).

**Results:**

Initial poor patient performance status, presence of liver metastases and detectable ^mut^*KRAS* ctDNA all correlated with worse OS after adjusting for the different biomarkers of interest. Objective response at 8 weeks also correlated with OS (*P* = 0.026). Plasma biomarkers measured during treatment and prior to the first response assessment identified ≥10% decrease in albumin at 4 weeks predicted for worse OS (HR 4.75, 95% CI 1.43–16.94, *P* = 0.012), while any association of longitudinal evaluation of ^mut^*KRAS* ctDNA with OS was unclear (*β* = 0.024, *P* = 0.057).

**Conclusions:**

Readily measurable patient variables can aid the prediction of outcomes from combination chemotherapy used to treat metastatic PDAC. The role of ^mut^*KRAS* ctDNA as a tool to guide treatment warrants further exploration.

**Clinical trial registration:**

ISRCTN71070888; ClinialTrials.gov (NCT03529175).

## Background

Patients diagnosed with metastatic pancreatic ductal adenocarcinoma (PDAC) generally have very poor overall survival, even with combination systemic therapy [1]. Nab-paclitaxel combined with gemcitabine (nabP+gemcitabine) is an international standard of care for metastatic PDAC, albeit offering modest improved outcomes for treated patients. The MPACT international registration randomised trial compared the efficacy of nabP+gemcitabine with gemcitabine alone and reported an overall response rate (ORR) of 23% [2] and median overall survival (OS) of 8.7 months [3] with the combination regimen. NabP + gemcitabine chemotherapy was associated with a significant risk of toxicities, with 50% of treated patients experiencing a serious adverse event and 38% having grade 3 or higher neutropenia. In 431 patients randomised to receive nabP+gemcitabine, 11 (3%) failed to start chemotherapy, while the median duration of treatment was 3.9 months, ranging between 0.1 and 21.9 months; 35% of patients were alive at 12 months.

The UK SIEGE randomised phase 2 trial compared sequential scheduling of nabP + gemcitabine administered 24 h apart, with standard delivery of the 2 drugs on the same day, as a potential strategy to improve outcomes in patients with metastatic PDAC [4]. Although ORR was higher in the sequential scheduling arm compared with standard concomitant drug delivery (51% versus 31%, *P* = 0.023), the OS in both arms was not statistically different (10.1 and 8.2 months, *P* = 0.70). One-year OS was 32% (95% CI 25–40%): remarkably similar to the MPACT trial population. The median number of 4-weekly cycles of chemotherapy delivered in the SIEGE trial was 3 (range 0–12) in the concomitant and 4 (range 0–24) in the sequential arm. In this trial, 25 (18%) treated patients failed to reach their first response assessment at 8 weeks, which, in 76% of cases, was due to rapidly progressing disease.

Both the MPACT and SIEGE trials demonstrate that, despite median OS times of 8–10 months, wide variation in individual outcomes is associated with the treatment of patients with metastatic PDAC. While chemotherapy currently represents the only treatment option associated with an extension in OS, treatment-related toxicity, impact on quality of life and limited survival gains means that selecting appropriate patients for intervention is challenging. Reliable prognostic markers, as well as markers predictive of response to chemotherapy, could potentially aid patient management but are currently lacking [5, 6]. In clinical practice, poor performance status (using either the Eastern Cooperative Oncology Group [ECOG PS] or Karnofsky [KPS] scales) and the presence of liver metastases are known to predict for poor disease outcomes [3, 7], but also characterise the majority of patients diagnosed with metastatic PDAC. The Lewis blood group antigen, carbohydrate antigen 19–9 (CA19–9) is the most well-established circulating tumour marker associated with PDAC, with higher blood levels generally associated with poorer survival [3, 8]. However, while frequently measured, it is rarely used to guide treatment decisions. In a retrospective study, the MPACT investigators demonstrated that any decrease in pre-treatment CA19-9 at week 8 of chemotherapy treatment was an early predictor of efficacy with nabP+gem [9]. Since most patients with metastatic PDAC will have repeat imaging to assess response at 8 weeks, measuring CA19-9 at the same time point as imaging offers limited additional clinical utility. However, they also showed that CA19-9 decrease identified more patients with survival benefit than radiological response at an 8-week assessment time point and differentiated those patients with CT-defined stable disease likely to do well. Blood samples were not collected at earlier timepoints within the MPACT trial, so the predictive value of CA19-9 changes earlier than 8 weeks could not be evaluated.

Alternative, readily measurable, soluble protein biomarkers have been proposed as showing potential prognostic significance when managing patients with both resectable and metastatic PDA. These mainly focus on markers of systemic inflammation with potential prognostic significance and include C-reactive protein (CRP) and albumin, as well as the neutrophil:lymphocyte ratio [10–12]. The modified Glasgow Prognostic score (mGPS) was validated as an inflammation-based prognostic score combining both CRP and albumin blood levels and has been reported to offer greater prognostic value compared with respective individual proteins [13–15]. There is also growing interest in the role of circulating tumour DNA (ctDNA) as a tool to aid PDAC management, although currently most data are based on limited retrospective patient series [16–18]. While there is general consensus that ctDNA has prognostic value, with higher levels correlating with poorer survival, few studies have explored its role in predicting for treatment outcomes. A German team measured plasma levels of mutant KRAS (^mut^*KRAS*) ctDNA weekly for the first 4 weeks after starting gemcitabine chemotherapy and reported that a decrease in ^mut^*KRAS* ctDNA was an early indicator of response to therapy, which was not seen for protein markers, including CA19-9 [19].

We used data and samples collected from patients recruited to the UK NIHR portfolio multi-centre SIEGE trial to explore potential clinical, protein and molecular circulating biomarkers measured prior to initiating nabP+gemcitabine chemotherapy for their ability to predict for treatment response and OS. We also explored serial circulating biomarker measurements during treatment, up to the time point of the first CT scan RECIST (Response Evaluation Criteria in Solid Tumours) response assessment performed at 8 weeks, for their ability to predict for treatment outcomes.

## Methods

### Patient population

Data from the SIEGE (Scheduling nab-paclItaxEl with GEmcitabine) trial has been published elsewhere [4]. In brief, patients aged ≥18 years with histologically-, or cytologically-confirmed metastatic PDAC and previously-untreated measurable metastatic disease, with KPS ≥ 70% were eligible for this trial. Other inclusion criteria included haemoglobin ≥100 g/L, platelets ≥100 × 10^9^/L, white blood cell count ≥3 × 10^9^/L, aspartate aminotransferase and/or alanine aminotransferase ≤2.5x upper limit of normal (ULN), bilirubin <1.5 × ULN, creatinine ≤1.5 × ULN. The SIEGE trial protocol (ISRCTN71070888) was approved by the Northern Ireland Research Ethics Committee 3 and was performed in accordance with the Declaration of Helsinki and the EU Clinical Trials Directive 2001/20/EC. All patients provided written informed consent. Grant funding for this investigator-initiated study was provided by Celgene Sarl. The funder had no role in the study design, data collection, analysis, interpretation, or writing of the report. Patients were randomised 1:1 using a random block method to receive six cycles of 4-weekly nabP+gemcitabine, administered either concomitantly (nabP 125 mg/m^2^ 30 min I.V. infusion immediately followed by gemcitabine 1000 mg/m^2^ I.V. infusion on days 1, 8 and 15 of a 28-day cycle), or sequentially: the same regimen, but gemcitabine administered on days 2, 9 and 16, starting 24 h after commencing nabP. Patients benefiting from treatment could continue beyond six cycles, at the investigator’s discretion.

### Procedures

Patients were assessed clinically prior to randomisation and at the start of each treatment cycle, then 4-weekly until disease progression and then 3-monthly. Imaging was performed at baseline and every 8 weeks until disease progression to assess objective response rate (ORR), using RECIST version 1.1. Provision of a pre-treatment tumour biopsy within 12 weeks of randomisation was mandatory. Routine blood samples required prior to treatment and each cycle of chemotherapy included full blood count (haemoglobin, white cell count, neutrophil count and platelet count) and biochemistry (including serum albumin and CRP). ECOG PS was recorded prior to starting on treatment only. The lymphocyte count was not recorded. Research blood samples to measure ctDNA were collected pre-randomisation and prior to cycle 2, after 4 weeks of treatment.

### Circulating tumour DNA measurement

Archival tumour tissue acquired within 3 months of trial entry was obtained for all patients. DNA from formalin-fixed paraffin-embedded (FFPE) tumour samples were extracted using QIAamp FFPE DNA Tissue kit (QIAGEN, Hilden, Germany). Plasma samples were collected at two timepoints within 21 days of starting chemotherapy: following study registration (baseline) and prior to cycle 1 day 1 chemotherapy administration. Plasma was collected prior to chemotherapy being administered on cycle 1 day 8, cycle 1 day 15 and cycle 2 day 1. For those patients randomised to sequential delivery of nabP 24 h before gemcitabine, plasma was also collected prior to administering chemotherapy on cycle 1 day 2, 9 and 16. Plasma was separated manually, aliquoted into microtubes and frozen at −80 °C. Circulating free (cf) DNA was subsequently extracted from available plasma samples using the QIASymphony Circulating DNA kit (QIAGEN). The number of copies of DNA per ml plasma in this set of samples varied between 795 and 972,828, with an average of 7333 copies per ml.

FFPE samples were tested for alterations on *KRAS* gene hotspot codons 12 and 13 using Fluidigm technology for Next-Generation Sequencing library preparation [18] and sequenced on a MiSeq (Illumina, San Diego, USA). Results were analysed with the ampliconseq pipeline (https://github.com/crukci-bioinformatics/ampliconseq) formerly developed to explore mutational processes in ovarian carcinomas [20], which uses GATK HaplotypeCaller [21] and VarDict [22] to call single nucleotide variants (SNVs) in target amplicons and models the background substitution noise at each amplicon position to filter SNV calls that are likely to be false positives. The plasma from patients with tumours containing alterations in *KRAS* hotspot codons 12 and 13 was analysed for ctDNA measurements of these alterations. ^mut^*KRAS* ctDNA was measured using droplet digital PCR (QX200 Bio-Rad, Hercules, USA) using the correspondent Bio-Rad mutation detection assays. Quanta soft v1.7.4 (Bio-Rad) was used to extrapolate the ddPCR results. The fractional abundance of mutant DNA was used to express the level of ctDNA in the plasma samples (see Supplementary Tables 1–3 for details of *KRAS* mutation detection and fractional abundance measurements).

### Statistical analysis

The following protein biomarkers were explored: CA19-9 (using three different cut-offs utilised by the IMPACT trial investigators [9]: above or below the upper limit of normal [ULN], above or below 200 U/ml and above or below 1000 U/ml), albumin (above or below 35 g/l, chosen as being the ULN), CRP (above or below 10 mg/l) and neutrophil count (above or below ULN). The mGPS was calculated based on individual CRP and albumin measurements and scored as either 0 (CRP ≤ 10 mg/l and any albumin level), 1 (CRP > 10 mg/l and albumin ≥35 g/l), or 2 (CRP > 10 mg/l and albumin <35 g/l) [12]. For ^mut^*KRAS* ctDNA, fractional abundance ≥1 was defined as detected in plasma and the maximum value of fractional abundance of the two samples collected prior to starting chemotherapy was used as the baseline value. Plasma samples for ^mut^KRAS ctDNA measurement were collected during the first 4 weeks of treatment, no samples were collected beyond the start of cycle 2 chemotherapy.

Results are presented as the mean ± standard deviation, median (range) for continuous variables, or percentage for category variables, wherever appropriate. The Association of the biomarkers at baseline (prior to starting chemotherapy) with OS was assessed using the Cox proportional-hazards model. To further evaluate the independent prognostic effect of ^mut^*KRAS* ctDNA on survival, multivariable Cox proportional hazard models adjusting different sets of variables were used. The Kaplan–Meier estimator was used to estimate the survival probability of patients stratified by objective tumour response at week 8 and ^mut^*KRAS* ctDNA detection status at baseline. To investigate the predictive effect of longitudinal biomarker alterations, the relationship of change from pre-treatment at week 4 and week 8 of different biomarkers with objective response at week 8, death within 1 year and OS were assessed using logistic regression or the Cox proportional-hazards model, adjusting for SIEGE trial treatment arm, where appropriate. To achieve a balanced frequency between categories, a different cut-off point for the decrease from baseline in CA19-9 (50%), CRP (50%), neutrophils (30%), and albumin (10%) was chosen. A sensitivity analysis was performed to investigate the longitudinal evaluation of ctDNA with OS using a joint model, details of which are provided in the Supplementary materials. All analyses were performed in R 4.1.0.

## Results

### Patients

Between March 2014 and March 2016, 146 patients with metastatic PDAC were recruited to the SIEGE trial (75 concomitant arm, 71 sequential arm) at 19 UK centres. The treatment arms were well-balanced for the covariates of interest (Table 1). At the start of treatment, there was a roughly even split of patients being either ECOG PS 0 or 1, although five patients (3%) had dropped their PS to 2 between the time of trial registration and starting treatment. At baseline, 86% of patients had CA19-9 levels >ULN and these were sub-divided into 3 categories: 86% >37 U/ml (ULN), 77% >200 U/ml, 62% >1000 U/ml; 27% of patients had raised neutrophil counts, 23% patients had lower than normal albumin and 51% patients had CRP > 10 mg/l. The proportions of patients in each of the mGPS categories were: 0 = 49%, 1 = 33%, 2 = 18%.

**Table 1:** Baseline characteristics of patients recruited to the SIEGE trial.

	Sequential arm (*N* = 71)	Concomitant arm (*N* = 75)	Total (*N* = 146)
Age	63.45 (8.32)	65.97 (8.16)	64.75 (8.31)
Sex			
Male	43/71 (60.6%)	40/75 (53.3%)	83/146 (56.8%)
Female	28/71 (39.4%)	35/75 (46.7%)	63/146 (43.2%)
Liver metastases present			
No	11/71 (15.5%)	13/75 (17.3%)	24/146 (16.4%)
Yes	60/71 (84.5%)	62/75 (82.7%)	122/146 (83.6%)
ECOG performance status			
0	30/70 (42.9%)	33/75 (44%)	63/145 (43.4%)
1	39/70 (55.7%)	38/75 (50.7%)	77/145 (53.1%)
2	1/70 (1.4%)	4/75 (5.3%)	5/145 (3.4%)
CA19-9 (37 U/ml)			
≤37 U/ml	10/69 (14.5%)	10/70 (14.3%)	20/139 (14.4%)
>37 U/ml	59/69 (85.5%)	60/70 (85.7%)	119/139 (85.6%)
CA19-9 (200 U/ml)			
≤200 U/ml	18/69 (26.1%)	14/70 (20%)	32/139 (23%)
>200 U/ml	51/69 (73.9%)	56/70 (80%)	107/139 (77%)
CA19-9 (1000 U/ml)			
≤1000 U/ml	31/69 (44.9%)	22/70 (31.4%)	53/139 (38.1%)
>1000 U/ml	38/69 (55.1%)	48/70 (68.6%)	86/139 (61.9%)
Albumin			
<35 g/L	18/71 (25.4%)	16/75 (21.3%)	34/146 (23.3%)
≥35 g/L	53/71 (74.6%)	59/75 (78.7%)	112/146 (76.7%)
C-reactive protein			
≤10 mg/l	32/70 (45.7%)	37/72 (51.4%)	69/142 (48.6%)
>10 mg/l	38/70 (54.3%)	35/72 (48.6%)	73/142 (51.4%)
mGPS			
0	32/70 (45.7%)	37/72 (51.4%)	69/142 (48.6%)
1	25/70 (35.7%)	22/72 (30.6%)	47/142 (33.1%)
2	13/70 (18.6%)	13/72 (18.1%)	26/142 (18.3%)
Neutrophils			
≤1*ULN	48/71 (67.6%)	59/75 (78.7%)	107/146 (73.3%)
>1*ULN	23/71 (32.4%)	16/75 (21.3%)	39/146 (26.7%)
^mut^*KRAS* ctDNA			
Detected	6/12 (50%)	8/11 (72.7%)	14/23 (60.9%)
Not detected	6/12 (50%)	3/11 (27.3%)	9/23 (39.1%)

*ULN*   upper limit of normal.

Twenty-three patients had sufficient pre-treatment tumour tissue as well as plasma DNA available to identify and quantify *KRAS* mutations, 14 of the 23 (61%) patients had ^mut^*KRAS* ctDNA detected (8 in the concomitant arm, 6 in the sequential arm). The mean fractional abundance was 25.51, ranging from 2.26 to 69.30. The *KRAS* variants identified were: G12D (*n* = 15), G12V (*n* = 4), G12R (*n* = 2), G12C (*n* = 1) and G61H (*n* = 1) (see also Supplementary Tables S1 and S2).

The primary end point of the SIEGE trial was the objective response rate, and patients were evaluable for response if they underwent radiological reassessment of measurable disease at approximately 8 weeks after starting chemotherapy. Twenty-nine patients failed to have radiological tumour assessment at 8 weeks: four patients who were randomised did not start chemotherapy: one patient in the concomitant arm progressed rapidly and died within 1 month of enrolment; two patients in the sequential arm withdrew consent; one patient in the sequential arm was subsequently found to be ineligible. Nineteen (13%) patients starting chemotherapy were confirmed to have progressed before their 8-week assessment, seven (5%) of whom had died.

### Overall survival correlations

Table 2 shows the univariate and multiple Cox regression results for the association of baseline biomarkers with OS. On univariate analysis, the following variables were significantly associated with a worse OS outcome: patients with liver metastases (HR = 1.78, 95% CI 1.09–2.92, *P* = 0.022), ECOG PS ≥ 1 (for PS1, HR = 1.66, 95% CI 1.15–2.40, *P* = 0.007; for PS2, HR = 4.15, 95% CI 1.60–10.74, *P* = 0.003), albumin <35 g/L (HR = 2.23, 95% CI 1.45–3.44, *P* < 0.001), CRP > 10 mg/l (HR = 1.62, 95% CI 1.13–2.32, *P* = 0.009), mGPS score ≥1 (for mGPS1, HR = 1.37, 95% CI 1.37–2.06, *P*  =  0.123; for mGPS2, HR = 2.46, 95% CI 1.49–4.06, *P*  < 0.001), ANC > ULN (HR = 2.06, 95% CI 1.36–3.10, *P* < 0.001) and detectable ctDNA (HR = 5.26, 95% CI 1.63–17.04, *P* = 0.006). As only a small number of patients had measurable plasma ^mut^*KRAS* ctDNA, this variable was not included in the multiple regression model. After including all the other variables of interest, only ECOG PS and liver metastasis remained significant in the regression model. All ECOG PS2 patients died within 1 year. Of note, 14% of patients had a baseline CA19–9 measurement of ≤37 U/mL (ULN), while none of the different cut-off values used for CA19-9 correlated with OS in our models.

**Table 2:** Univariate and multiple Cox regression results for the association of baseline markers with overall survival.

Variable	Univariate		Multivariate					
	HR (95% CI)	*P*	HR (95% CI)	*P*	HR (95% CI)	*P*	HR (95% CI)	*P*
Liver metastases present (ref no)								
Yes	1.78 (1.09, 2.92)	0.022	2.08 (1.22, 3.54)	0.007	1.98 (1.17, 3.35)	0.011	2.00 (1.17, 3.42)	0.011
ECOG performance status (ref 0)								
1	1.66 (1.15, 2.40)	0.007	1.64 (1.10, 2.46)	0.016	1.67 (1.12, 2.48)	0.011	1.72 (1.14, 2.58)	0.009
2	4.15 (1.60, 10.74)	0.003	1.71 (0.55, 5.28)	0.352	1.74 (0.57, 5.36)	0.332	1.83 (0.59, 5.65)	0.294
Albumin (ref ≥ 35 g/L)								
<35 g/L	2.23 (1.45, 3.44)	<0.001	2.32 (0.89, 6.09)	0.087	2.32 (0.88, 6.09)	0.087	2.26 (0.86, 5.91)	0.097
C-reactive protein (ref ≤ 10 mg/l)								
>10 mg/l	1.62 (1.13, 2.32)	0.009	0.87 (0.31, 2.44)	0.799	0.91 (0.33, 2.54)	0.861	0.90 (0.32, 2.52)	0.844
mGPS (ref 0)								
1	1.37 (0.92, 2.06)	0.123	1.39 (0.47, 4.14)	0.555	1.33 (0.45, 3.97)	0.605	1.36 (0.45, 4.05)	0.584
2	2.46 (1.49, 4.06)	<0.001	–		–		–	
Neutrophils (ref ≤ 1*ULN)								
>1*ULN	2.06 (1.36, 3.10)	<0.001	1.40 (0.87, 2.25)	0.168	1.39 (0.86, 2.23)	0.177	1.39 (0.86, 2.24)	0.174
CA19-9 (1000) (ref ≤ 1000 U/ml)								
>1000 U/ml	1.18 (0.80, 1.75)	0.401					1.02 (0.67, 1.56)	0.934
CA19-9 (200) (ref ≤ 200 U/ml)								
>200 U/ml	1.30 (0.81, 2.09)	0.271			1.23 (0.75, 2.01)	0.408		
CA19-9 (37) (ref ≤ 37 U/ml)								
>37 U/ml	1.37 (0.79, 2.38)	0.257	1.29 (0.72, 2.30)	0.397				
^mut^*KRAS* ctDNA (ref not detected)								
Detected	5.26 (1.63, 17.04)	0.006						

*HR* hazard ratio, *CI* confidence interval.

All models were adjusted for the treatment arm; ^mut^KRAS ctDNA was not included in the multivariate model due to the small sample size.

### Correlation of 8-week objective response with OS

**Fig. 1:**
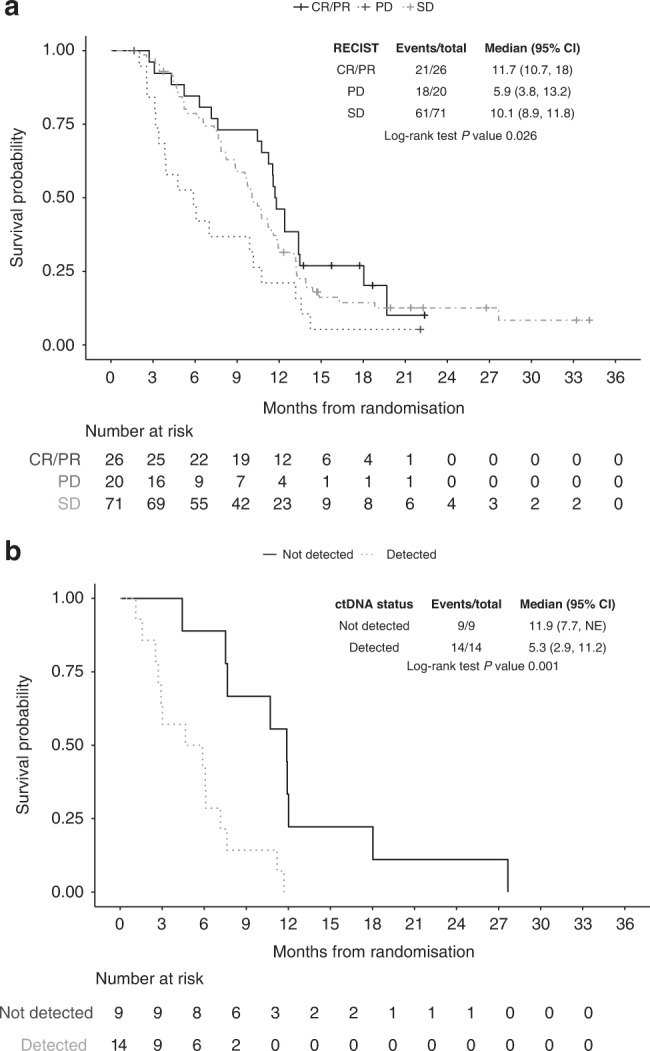
Overall survival Kaplan-Meier curves stratified by RECIST response and ^mut^*KRAS* ctDNA status. **a** Overall survival Kaplan–Meier curves, stratified by RECIST response at week 8 of chemotherapy. CR   complete response, PR   partial response, SD   stable disease, PD   progressive disease. **b** Overall survival Kaplan–Meier curves, stratified by ^mut^*KRAS* ctDNA detected prior to starting chemotherapy. NE  not estimated.

Kaplan–Meier analysis (Fig. 1a) of the 117 patients who underwent a radiological assessment at week 8 demonstrated that RECIST response at 8 weeks predicted for OS (log-rank *P* = 0.026). Twenty-six (22%) patients (95% CI 15%–31%) had documented objective response at this initial assessment time point compared with 48 (41%, 95% CI 32–50%) patients who achieved an objective response during the course of the trial overall (Supplementary Table S4). This was due to 24 patients with stable disease at week 8 achieving a subsequent objective response: 71 (61%) patients had stable disease at 8 weeks, 49 (42%) patients had stable disease as their best response, while 20 (17%) patients had progressive disease recorded for both response assessments. The disease control rates were therefore almost identical: 83% at week 8 and 82% overall.

Patients achieving an objective response at 8 weeks had the longest survival times, with median OS 11.7 (95% CI 10.7–18) months, compared with 10.1 (95% CI 8.9–11.8) months for stable disease and 5.9 (95% CI 3.8–13.2) months for patients with disease progression (*P* < 0.001). During the follow-up period of the trial, 21 (81%) of the 26 patients who responded to chemotherapy at 8 weeks had died, compared with 87% of patients who had either stable disease (61/71 patients) or disease progression (18/20 patients) at 8 weeks.

### Exploration of baseline ^mut^*KRAS* ctDNA association with OS

All the patients who had ^mt^*KRAS* ctDNA measurements available have subsequently died. OS analysis showed that patients with undetectable ^mut^*KRAS* ctDNA prior to starting treatment had significantly longer survival times compared to those with detectable ^mut^*KRAS* ctDNA (median OS 11.9 versus 5.3 months, *P* = 0.001; Fig. 1b). Table 3 summarises the multiple Cox regression results for the association of baseline ^mut^*KRAS* ctDNA and OS, adjusting for different variables. The OS HR for detected versus not detected ^mut^*KRAS* ctDNA ranged from 7.59 (95% CI 2.22–25.9, *P* = 0.001) to 83.6 (95% CI 5.71–1224, *P* = 0.001) and remained significant after incrementally adjusting for patient demographics (age and sex), tumour characteristics (ECOG PS and liver metastasis) and protein biomarkers (mGPS and CA19-9).

**Table 3:** Multiple Cox regression results for the association of baseline ^mut^KRAS ctDNA and overall survival, adjusting for different markers.

	Model 1		Model 2		Model 3	
Variable	HR (95% CI)	*P*	HR (95% CI)	*P*	HR (95% CI)	*P*
^mut^*KRAS* ctDNA (ref: not detected)						
Detected	7.59 (2.22, 25.90)	0.001	22.60 (3.11, 164.27)	0.002	83.59 (5.71, 1223.90)	0.001
Age	1.05 (0.97, 1.14)	0.206	1.05 (0.95, 1.16)	0.352	1.07 (0.97, 1.19)	0.191
Sex (ref: male)						
Female	0.22 (0.07, 0.74)	0.014	0.15 (0.03, 0.81)	0.027	0.03 (0.00, 0.31)	0.004
ECOG performance status (ref: 0)						
1			8.82 (2.19, 35.63)	0.002	12.10 (1.66, 88.09)	0.014
2			3.77 (0.70, 20.31)	0.123	1.32 (0.18, 9.70)	0.783
Liver metastases present (ref: no)						
Yes			1.56 (0.19, 12.95)	0.680	0.51 (0.05, 5.60)	0.582
mGPS (ref: 0)						
2					42.34 (2.28, 785.40)	0.012
1					0.30 (0.05, 1.80)	0.187
CA19-9 (ref: ≤1000)						
>1000					1.51 (0.26, 8.65)	0.645

*HR* hazard ratio, *CI* confidence interval, *ref* reference group.

Model 1: ^mut^KRAS ctDNA + treatment + age + sex.

Model 2: variables in model 1 + ECOG performance status + Liver metastases.

Model 3: variables in model 2 + mGPS + CA19-9.

ANC was not included in model 3 due to the large SE.

### Longitudinal biomarker changes

**Fig. 2:**
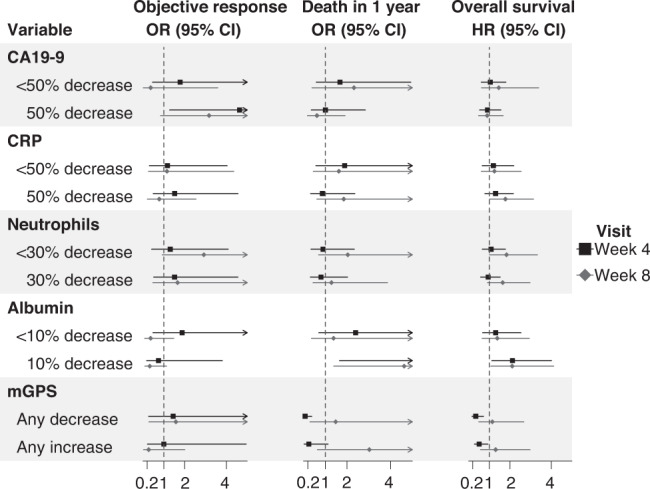
Forest plot of logistic and Cox regression results for the association of changes in circulating biomarkers at week 4 and week 8 with the objective response at week 8, death within 1 year and overall survival. Note that the CA19-9, CRP, ANC (absolute neutrophil count) and albumin were compared with no change or any decrease. The mGPS was compared with no change.

Figure 2 and Supplementary Table S5 present the logistic and Cox regression results for the association of changes in putative biomarkers (CA19-9, albumin, CRP and mGPS) measured up to 8 weeks with objective response, death within 1 year and OS. Neutrophil count was omitted because of the confounding myelosuppressive effect of cytotoxic chemotherapy. A ≥ 50% decrease in CA19-9 (compared to no change or any increase) at week 4 was significantly associated with an objective response at week 8 (HR 4.63, 95% CI 1.28–20.15, *P* = 0.026), but this was not true for death within 1 year or OS. A ≥ 10% decrease in albumin (vs no change or any increase) at both week 4 and week 8 was significantly associated with increased risk of death within 1 year (HR 5.76, 95% CI 1.70–21.68, *P* = 0.006 and HR 4.75, 95% CI 1.43–16.94, *P* = 0.012), as well as OS (HR 2.17, 95% CI 1.15–4.09, *P* = 0.017 and HR 2.13, 95% CI 1.09–4.15, *P* = 0.027). Longitudinal changes in mGPS score were inconsistent: while any decrease in mGPS score at week 4 was significantly associated with a lower risk of death within 1 year (HR 0.07, 95% CI 0.01–0.38, *P* = 0.004) and OS (HR 0.35, 95% CI 0.17–0.72, *P* = 0.004), an increase in mGPS score was also associated with lower risk of OS (HR 0.51, 95% CI 0.28–0.93, *P* = 0.028). Changes in mGPS score at 8 weeks were not significantly associated with any efficacy outcomes.

**Fig. 3:**
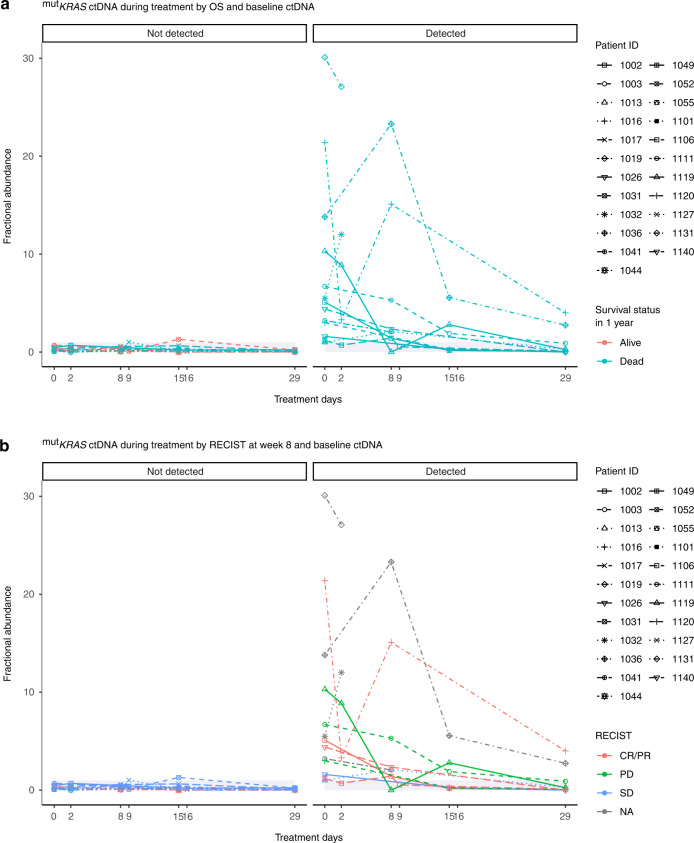
^mut^*KRAS* ctDNA measurements during the first 4 weeks of chemotherapy treatment. Longitudinal ctDNA measurements for cases are separated by whether ctDNA was detected or not detected at baseline (pre-treatment) and different colours identify survival status at 1 year (**a**) or RECIST response at week 8 (**b**). The grey-shaded area demonstrates the fractional abundance of ctDNA <1.

Longitudinal measurement of ^mut^*KRAS* ctDNA fractional abundance demonstrated a steep drop after 4 weeks of treatment for patients with detectable ^mut^*KRAS* ctDNA at baseline (Fig. 3). In patients where ctDNA was undetectable at baseline, there was no discernible change in fractional abundance. All three patients who were alive after 1 year had undetectable ctDNA at baseline, and all three patients with progressive disease at week 8 had detectable ctDNA at baseline.

Eighteen of the 23 patients who had ^mut^*KRAS* ctDNA detectable at baseline had plasma samples available for ctDNA analysis at 4 weeks (Supplementary Table S6). At 4 weeks, there was a decrease in ctDNA in 17 cases and in 8 of these cases, the ctDNA was undetectable. There was no significant correlation between changes in ^mut^*KRAS* ctDNA from baseline during the first 4 weeks of treatment and objective response at week 8, survival status at 1 year, or OS. In the sensitivity analysis, the longitudinal changes in ctDNA were not significant, either with (*β* = 0.024, *P* = 0.057) or without (*β* = −0.012, *P* = 0.337) adjustment of baseline ctDNA status (Supplementary Table S7).

## Discussion

In this retrospective analysis of the SIEGE trial dataset, radiological response assessment at 8 weeks was a good surrogate marker for OS in patients with metastatic PDAC treated with nabP+gem chemotherapy, primarily by selecting out those patients with worst survival times, most of whom demonstrated disease progression at their first imaging assessment. The primary aim of this study was to determine whether other easily accessible and measurable markers could add further benefit compared with standard imaging to predict for efficacy outcomes, when measured either prior to starting treatment or within the first 8 weeks of treatment.

Consistent with many other published studies, we identified that patient ECOG PS and the presence or absence of liver metastases prior to initiating chemotherapy were the strongest predictors of OS. In the SIEGE patient cohort, CA19-9 concentration did not correlate with OS, despite using three different cut-off values. While CA19-9 is considered a clinically useful biomarker for pancreatic cancer, it is far from ideal, given that about 10% of the general population does not express the Lewis blood antigen, while false positive results can be generated by other conditions commonly occurring alongside PDAC, including pancreatitis, cholestasis, diabetes and liver cirrhosis, as well as other cancers. The inability to demonstrate CA19-9 as a prognostic marker in the SIEGE trial serves as an example of its unreliability and hence limited clinical utility.

Many observational studies have generated a wealth of data across multiple tumour types on the prognostic value of the systemic inflammatory response. In the SIEGE study, elevated circulating markers of inflammation (CRP, neutrophils) and low albumin levels all correlated with shorter OS, although none of these were significant in a multivariate regression model. Furthermore, mGPS—a score which combines both albumin and CRP measures—did not offer greater prognostic value than the individual components. While advocates have called for the evaluation of combined prognostic scores in prospective randomised clinical trials [13], mGPS has not yet found its way into the clinic. A recent meta-analysis of 25 publications including 4,629 patients with PDAC concluded that elevated mGPS correlated with poor OS (HR 1.92, 95% CI 1.6–2.3, *P* < 0.002) [23]. However, there were only three prospective studies included and the PDAC population was of mixed disease stages. Furthermore, no attempt was made to correlate albumin or CRP independently with outcomes. We hypothesise that mGPS might not add significant value beyond the individual inflammatory markers used to generate the combined score. Our findings that mGPS is not an independent prognostic marker of OS in the prospective SIEGE trial suggest that further assessment is still needed before incorporating it into routine clinical practice.

The SIEGE study explored the measurement of ^mut^*KRAS* ctDNA as a biomarker of response to chemotherapy in patients with metastatic PDAC. Although the study mandated the provision of a tumour biopsy at study entry, in only 23 of 146 patients entering the study was there both sufficient tumour and plasma to be able to identify and quantify ^mut^*KRAS* ctDNA in both sources. Tissue samples from all 23 patients had detectable *KRAS* mutations in codons 12 and 13. Of those 23 patients with ^mut^*KRAS*-positive tumours, 14 had detectable amounts of ^mut^*KRAS* ctDNA in plasma collected prior to receiving chemotherapy. This detection rate of 61% is consistent with published studies, with rates likely varying due to different detection methodologies used [16–18]. Over 90% of PDACs harbour a KRAS mutation, and in 90% of those, one of three common polymorphisms: G12D, G12V or G12R will be identified. Our data (65%, 17% and 9%, respectively) reflected this. CtDNA analytical methods have focused on PCR technology which is less costly than next-generation sequencing while limiting the number of mutations interrogated. Droplet digital PCR (ddPCR) has exceptional sensitivity and only requires a low starting concentration of DNA template. Our findings add to accumulating evidence that detection of ^mut^*KRAS* ctDNA per se is a prognostic marker of poor outcome: the presence of detectable ^mut^*KRAS* ctDNA is associated with worse survival [16–18]. The high hazard ratios (between 7.59 and 83.6) for detected versus not detected at baseline in our study will be, at least in part, due to the small sample size here. However, detectable ^mut^*KRAS* ctDNA remained significant after adjusting for patient demographics, tumour characteristics and other protein biomarkers, suggesting this is likely to be an independent prognostic marker in patients with metastatic PDAC.

In this study, we were interested to explore kinetics of readily accessible variables within the first 8 weeks of treatment, given that a high proportion of metastatic PDAC (in the SIEGE trial, 13%) patients deteriorate rapidly and fail to reach their first objective response assessment usually planned for after 8 weeks of treatment. Of the protein markers of interest (CA19-9, albumin, CRP), only a decrease in albumin levels measured both at 4 and 8 weeks consistently correlated with worse OS. Changes in CRP did not correlate with the response or survival outcomes and the consequential calculated mGPS was inconsistent. In this dataset, we found that a decrease in CA19-9 at week 4 correlated with ORR at week 8, but not with ultimate survival outcomes, arguably the more important efficacy end point when treating metastatic PDAC.

The potential value of ^mut^*KRAS* ctDNA as a highly specific non-invasive tool in early response prediction and therapeutic monitoring in metastatic PDAC was highlighted recently by Kruger et al. [19] who measured ^mut^*KRAS* ctDNA in 54 patients with advanced (87% metastatic) PDAC receiving palliative, predominantly gemcitabine-based chemotherapy. The investigators correlated ctDNA in addition to protein biomarkers including CA19-9 and found that ^mut^*KRAS* ctDNA had high sensitivity and specificity for tissue *KRAS* mutation status. High baseline ^mut^*KRAS* ctDNA correlated with poor OS, while decreasing levels within the first 4 weeks of treatment correlated with response to treatment (albeit not reaching statistical significance), which was not seen with CA19-9 changes. Other recent studies support the potential role of ctDNA as a surveillance tool for patients with PDAC being monitored over longer time periods, measured in months and years [24, 25], with good correlation between ctDNA, CA19.9 expression and imaging over time.

Our own data suggest that longitudinal evaluation of ^mut^*KRAS* ctDNA within the first cycle of gemcitabine-based chemotherapy may be of limited additional value in predicting longer term outcomes relative to the value of initial baseline detection, which strongly correlates with OS: patients with detectable ^mut^*KRAS* ctDNA at baseline had significantly worse OS compared with patients with undetectable ^mut^*KRAS* ctDNA and none survived beyond 1 year. Of note, ctDNA levels in those patients whose ^mut^*KRAS* ctDNA was undetectable at baseline remained largely undetectable throughout the treatment period. Any absolute decrease (versus increase) or changes (versus no changes) in ctDNA at different timepoints during the first month of treatment were not associated with OS. This was also confirmed by the sensitivity analysis, which showed that the longitudinal evaluation of ctDNA was not associated with OS. Thus, while baseline ctDNA status was associated with OS, early ctDNA dynamic changes in the first month of treatment were not. Similar to our findings, Tolmeijer and colleagues [26] found a strong link between ctDNA at baseline and OS in a group of advanced castrate-resistant prostate cancer patients, but the changes from high to low ctDNA levels at 4 weeks of androgen receptor inhibition was not correlated with OS.

CtDNA can be measured using a variety of different analytical technologies, which may influence findings. We have applied ddPCR, while others have used targeted sequencing of a small number of key hotspot mutations [24], or broader whole exome sequencing [25]. The different trade-offs, including ctDNA detection rate, ability to track single versus multiple molecular alterations, as well as ease of access and cost implications, are important factors that need to be explored in order to best inform the application of ctDNA measurement in future patient management.

In summary, this study explored simple tools which might aid clinicians when considering prognosis and treatment options for patients with metastatic PDAC. Information that is already part of the standard practice—ECOG PS, presence of liver metastases and initial objective response—are the most powerful predictors of survival. Additional information provided by routine blood tests either prior to starting or during the first 8 weeks of treatment offered very little additional prognostic or predictive information, although a falling albumin during the 8 weeks after starting treatment correlated with poor OS. Despite a strong rationale for its application to PDAC, we found that mGPS performed poorly both in a prognostic and predictive capacity in the SIEGE trial patient cohort. On the other hand, our data supports growing evidence that detection of ^mut^*KRAS* ctDNA prior to starting treatment may provide useful prognostic information for clinicians and patients, and further studies of this technology are strongly recommended.

## Supplementary information

Supplementary Materials

## Data Availability

The SIEGE trial data is held by the Cambridge Clinical Trials Unit-Cancer Theme. Ownership of the data resides with the Trial Management Group (TMG). Access to the data can be requested and authorised by the TMG.

## Code availability

Scripts used to generate the results presented in this study are available from the author AD on reasonable request.
