# Supplementary material for: Clinical and biological markers predictive of treatment response associated with metastatic pancreatic adenocarcinoma
Source: Br J Cancer. 2023 Feb 22;128(9):1672–80. doi: 10.1038/s41416-023-02170-9 (PMC10133256; doi:10.1038/s41416-023-02170-9)
Supplement: Supplementary file 1 — Supplementary Materials [file 41416_2023_2170_MOESM1_ESM.docx]

**Supplementary Materials**

**Joint model used to study ^mut^KRAS ctDNA kinetics**

To investigate the association of longitudinal evaluation of the ctDNA with the OS, we used a joint model for the longitudinal and time-to-event outcome^1^. The joint model consists of two sub-models: a linear mixed model for the longitudinal ctDNA and a model for the OS, linked by an association structure.

In the longitudinal modelling process, the ^mut^KRAS ctDNA fractional abundance was log-transformed due to its skew nature. A linear mixed model, with random intercept and slope, including time of ctDNA measurement since the treatment and its interaction with treatment arm in the fixed effect was applied. A compound symmetry correlation structure was assumed for the covariance of the random effects. Two models with and without baseline ctDNA detection status plus the treatment arm were fitted for the survival sub-model. The linear mixed model was fitted using lme4 (version 1.1-27.1) and the joint model was fitted using JM (version 1.4-8) package ^2,3^.

1. Papageorgiou G, Mauff K, Tomer A, Rizopoulos D. An Overview of Joint Modeling of Time-to-Event and Longitudinal Outcomes. *Annual Review of Statistics and Its Application*. 2019;6(1):223-240. doi:10/gk85cs

2. Bates D, Mächler M, Bolker B, Walker S. Fitting Linear Mixed-Effects Models Using lme4. *Journal of Statistical Software*. 2015;67:1-48. doi:10/gcrnkw

3. Rizopoulos D. JM: An R Package for the Joint Modelling of Longitudinal and Time-to-Event Data. *Journal of Statistical Software*. 2010;35:1-33. doi:10/ghv7n4

**Supplementary Table S1.** Summary of plasma ^mut^KRAS ctDNA detected at baseline and its fractional abundance

|  |  | Mean value ^a^ | | Maximum value ^b^ | |
| --- | --- | --- | --- | --- | --- |
| **Target** | **N** | **Mean (SD)** | **Median (min, max)** | **Mean (SD)** | **Median (min, max)** |
| KRAS_G12C | 1 | 13.55 (.) | 13.55 (13.55, 13.55) | 13.80 (.) | 13.80 (13.80, 13.80) |
| KRAS_G12D | 15 | 4.61 (7.92) | 1.26 (0.07, 29.40) | 5.23 (8.73) | 1.26 (0.08, 30.10) |
| KRAS_G12R | 2 | 4.65 (6.43) | 4.65 (0.11, 9.20) | 5.23 (7.17) | 5.23 (0.16, 10.30) |
| KRAS_G12V | 4 | 0.64 (0.66) | 0.44 (0.08, 1.60) | 0.71 (0.65) | 0.57 (0.08, 1.60) |
| KRAS_Q61H | 1 | 4.65 (.) | 4.65 (4.65, 4.65) | 5.50 (.) | 5.50 (5.50, 5.50) |

^a^ Mean value of two samples collected prior to starting chemotherapy for each patient.

^b^ Maximum value of two samples collected prior to starting chemotherapy for each patient.

**Supplementary Table S2.** Summary of tissue ^mut^KRAS ctDNA detected and its fractional abundance

| Target | N | Mean (SD) | Median (min, max) |
| --- | --- | --- | --- |
| KRAS_G12C | 1 | 69.30 (.) | 69.30 (69.30, 69.30) |
| KRAS_G12D (mean) | 14 | 25.35 (13.20) | 19.90 (2.50, 48.90) |
| KRAS_G12D (max) |  | 26.41 (14.50) | 20.00 (2.50, 50.20) |
| KRAS_G12R | 2 | 14.80 (3.25) | 14.80 (12.50, 17.10) |
| KRAS_G12V | 4 | 16.39 (10.31) | 18.20 (2.26, 26.90) |
| KRAS_Q61H | 1 | 45.00 (.) | 45.00 (45.00, 45.00) |

Note: for 3 patients (all in KRAS_G12D) with two tissue sample analysed, the max and average values are provided.

**Supplementary Table S3.** Summary of plasma ^mut^KRAS ctDNA at different time points and change from baseline

|  | Value | | |  | Change from baseline | | |
| --- | --- | --- | --- | --- | --- | --- | --- |
| Day ^a^ | N | Mean (SD) | Median (min, max) |  | N | Mean (SD) | Median (min, max) |
| Day 0 | 23 | 4.83 (7.60) | 1.26 (0.08, 30.10) |  | - | - | - |
| Day 2 | 10 | 5.31 (8.74) | 0.70 (0.00, 27.10) |  | 10 | -1.66 (6.27) | -0.12 (-18.10, 6.50) |
| Day 8 | 17 | 3.25 (6.32) | 0.57 (0.00, 23.30) |  | 17 | -0.81 (3.93) | -0.01 (-10.30, 9.50) |
| Day 9 | 3 | 0.55 (0.47) | 0.53 (0.09, 1.02) |  | 3 | 0.08 (0.80) | -0.07 (-0.64, 0.94) |
| Day 15 | 15 | 0.93 (1.51) | 0.23 (0.00, 5.56) |  | 15 | -1.94 (3.01) | -0.34 (-8.24, 1.14) |
| Day 16 | 2 | 0.12 (0.11) | 0.12 (0.05, 0.20) |  | 2 | -0.14 (0.16) | -0.14 (-0.25, -0.03) |
| Day 29 | 18 | 0.50 (1.09) | 0.07 (0.00, 4.00) |  | 18 | -3.43 (4.89) | -0.91 (-17.40, 0.05) |

^a^ Treatment Day, day 0 is baseline, day 29 is cycle 2 day 1.

**Supplementary Table S4**: Efficacy outcomes associated with the SIEGE trial patient population

|  | **Concomitant**  **(N=75)** | **Sequential**  **(N=71)** | **Overall**  **(N=146)** |
| --- | --- | --- | --- |
|  |  |  |  |
| Patients assessed for response | 61 | 56 | 117 |
| **Best overall response**  Partial response (PR) | 19 (31%) | 28 (50%) | 47 (40%) |
| Stable disease (SD) | 29 (48%) | 20 (36%) | 49 (42%) |
| Progressive disease (PD) | 13 (21%) | 7 (13%) | 20 (17%) |
|  |  |  |  |
|  |  |  |  |
| Overall response rate (CR+PR)  *p*-value=0.023 | 31%  (95%CI 20%-44%) | 52%  (95%CI 38%-65%) | 41%  (95%CI 32%-50%) |
|  |  |  |  |
| **Objective response at week 8** |  |  |  |
| Partial response (PR) | 10 (29%) | 16 (16%) | 26 (22%) |
| Stable disease (SD) | 38 (59%) | 33 (62%) | 71 (61%) |
| Progressive disease (PD) | 13 (12%) | 7 (21%) | 20 (17%) |
| Overall response rate (CR+PR)  *p*-value=0.105 | 16%  (95%CI 8%-28%) | 29%  (95%CI 17%-42%) | 22%  (95%CI 15%-31%) |
|  |  |  |  |
| **1 year survival** |  |  |  |
| Death | 52/75 | 47/71 | 99/146 |
| 95% CI | 69% (58%-79%) | 66% (54%-77%) | 68% (60%-75%) |
|  |  |  |  |
| **OS** |  |  |  |
| Death | 64/75 | 59/71 | 123/146 |
| Median OS (months) | 8.2  (95%CI 6.1–10.7) | 10.2  (95%CI 7.0–11.5) | 9.0  (95%CI 7.4-10.7) |

*Response was evaluated by investigators and central radiological review was performed for 10% of randomly selected patients with good concordance. Most discrepancies related to non-target lesions and did not affect key outcome measures. This is from the all patients randomised.

**Supplementary Table S5.** Logistic and Cox regression results for the association of changes in biomarkers from baseline at week 4 and week 8 with objective response at week 8, death within 1 year and OS

|  |  | Objective Response | | | Death in 1 year | | | Overall survival | | |
| --- | --- | --- | --- | --- | --- | --- | --- | --- | --- | --- |
| Variable | N | ORR (%) | OR (95% CI) | P | Numbers (%) | OR (95% CI) | P | Numbers (%) | HR (95% CI) | P |
| **CA19.9 - Week 4** |  |  |  |  |  |  |  |  |  |  |
| No change or any increase | 31 | 4 (13.3%) |  |  | 19 (61.3%) |  |  | 27 (87.1%) |  |  |
| < 50% decrease | 33 | 7 (22.6%) | 1.78 (0.46, 7.70) | 0.410 | 24 (72.7%) | 1.68 (0.59, 4.93) | 0.338 | 27 (81.8%) | 1.05 (0.62, 1.80) | 0.852 |
| ≥ 50% decrease | 31 | 11 (35.5%) | 4.63 (1.28, 20.15) | 0.026 | 19 (61.3%) | 1.01 (0.36, 2.84) | 0.989 | 27 (87.1%) | 0.91 (0.53, 1.56) | 0.737 |
| **CA19.9 - Week 8** |  |  |  |  |  |  |  |  |  |  |
| No change or any increase | 17 | 3 (17.6%) |  |  | 11 (64.7%) |  |  | 14 (82.4%) |  |  |
| < 50% decrease | 10 | 1 (10%) | 0.37 (0.02, 3.59) | 0.429 | 8 (80%) | 2.32 (0.39, 19.19) | 0.379 | 9 (90%) | 1.46 (0.63, 3.39) | 0.382 |
| ≥ 50% decrease | 47 | 18 (38.3%) | 3.17 (0.85, 15.71) | 0.111 | 25 (53.2%) | 0.62 (0.19, 1.90) | 0.410 | 38 (80.9%) | 0.90 (0.48, 1.67) | 0.732 |
| **CRP - Week 4** |  |  |  |  |  |  |  |  |  |  |
| No change or any increase | 51 | 11 (21.6%) |  |  | 33 (64.7%) |  |  | 42 (82.4%) |  |  |
| < 50% decrease | 16 | 4 (25%) | 0.94 (0.22, 3.48) | 0.925 | 13 (81.2%) | 2.37 (0.64, 11.50) | 0.228 | 14 (87.5%) | 1.41 (0.75, 2.65) | 0.285 |
| ≥ 50% decrease | 28 | 9 (33.3%) | 1.68 (0.57, 4.91) | 0.342 | 17 (60.7%) | 0.84 (0.33, 2.22) | 0.726 | 25 (89.3%) | 1.20 (0.73, 1.97) | 0.470 |
| **CRP - Week 8** |  |  |  |  |  |  |  |  |  |  |
| No change or any increase | 43 | 12 (27.9%) |  |  | 25 (58.1%) |  |  | 33 (76.7%) |  |  |
| < 50% decrease | 12 | 4 (33.3%) | 1.27 (0.29, 4.94) | 0.732 | 9 (75%) | 2.20 (0.56, 11.04) | 0.285 | 10 (83.3%) | 1.65 (0.81, 3.39) | 0.169 |
| ≥ 50% decrease | 22 | 5 (22.7%) | 0.74 (0.21, 2.40) | 0.631 | 15 (68.2%) | 1.57 (0.54, 4.86) | 0.417 | 20 (90.9%) | 1.51 (0.86, 2.63) | 0.151 |
| **ANC - Week 4** |  |  |  |  |  |  |  |  |  |  |
| No change or any increase | 44 | 8 (19%) |  |  | 29 (65.9%) |  |  | 37 (84.1%) |  |  |
| < 30% decrease | 33 | 8 (25%) | 1.31 (0.42, 4.09) | 0.640 | 21 (63.6%) | 0.89 (0.35, 2.33) | 0.818 | 28 (84.8%) | 1.09 (0.66, 1.78) | 0.739 |
| ≥ 30% decrease | 36 | 10 (27.8%) | 1.52 (0.52, 4.55) | 0.447 | 22 (61.1%) | 0.80 (0.32, 2.02) | 0.639 | 31 (86.1%) | 0.94 (0.58, 1.53) | 0.817 |
| **ANC - Week 8** |  |  |  |  |  |  |  |  |  |  |
| No change or any increase | 36 | 7 (19.4%) |  |  | 20 (55.6%) |  |  | 26 (72.2%) |  |  |
| < 30% decrease | 25 | 10 (40%) | 2.92 (0.93, 9.76) | 0.072 | 18 (72%) | 2.03 (0.70, 6.36) | 0.204 | 22 (88%) | 1.84 (1.01, 3.33) | 0.045 |
| ≥ 30% decrease | 23 | 7 (30.4%) | 1.66 (0.48, 5.77) | 0.420 | 14 (60.9%) | 1.28 (0.44, 3.86) | 0.651 | 21 (91.3%) | 1.65 (0.92, 2.97) | 0.095 |
| **ALBUMIN - Week 4** |  |  |  |  |  |  |  |  |  |  |
| No change or any increase | 14 | 3 (21.4%) |  |  | 5 (35.7%) |  |  | 12 (85.7%) |  |  |
| < 10% decrease | 41 | 14 (34.1%) | 1.88 (0.48, 9.42) | 0.391 | 23 (56.1%) | 2.29 (0.67, 8.60) | 0.197 | 33 (80.5%) | 1.27 (0.66, 2.46) | 0.478 |
| ≥ 10% decrease | 58 | 9 (16.4%) | 0.74 (0.18, 3.80) | 0.695 | 44 (75.9%) | 5.76 (1.70, 21.68) | 0.006 | 51 (87.9%) | 2.17 (1.15, 4.09) | 0.017 |
| **ALBUMIN - Week 8** |  |  |  |  |  |  |  |  |  |  |
| No change or any increase | 15 | 7 (46.7%) |  |  | 6 (40%) |  |  | 11 (73.3%) |  |  |
| < 10% decrease | 22 | 5 (22.7%) | 0.31 (0.07, 1.28) | 0.111 | 10 (45.5%) | 1.26 (0.33, 4.94) | 0.738 | 18 (81.8%) | 1.35 (0.63, 2.87) | 0.439 |
| ≥ 10% decrease | 50 | 12 (24%) | 0.36 (0.10, 1.22) | 0.096 | 38 (76%) | 4.75 (1.43, 16.94) | 0.012 | 43 (86%) | 2.13 (1.09, 4.15) | 0.027 |
| **mGPS - Week 4** |  |  |  |  |  |  |  |  |  |  |
| No change | 20 | 4 (21.1%) |  |  | 18 (90%) |  |  | 20 (100%) |  |  |
| Any decrease | 15 | 4 (26.7%) | 1.45 (0.27, 7.81) | 0.659 | 6 (40%) | 0.07 (0.01, 0.38) | 0.004 | 12 (80%) | 0.35 (0.17, 0.72) | 0.004 |
| Any increase | 30 | 5 (16.7%) | 1.00 (0.22, 4.94) | 0.997 | 21 (70%) | 0.24 (0.03, 1.13) | 0.100 | 26 (86.7%) | 0.51 (0.28, 0.93) | 0.028 |
| **mGPS - Week 8** |  |  |  |  |  |  |  |  |  |  |
| No change | 12 | 3 (25%) |  |  | 7 (58.3%) |  |  | 9 (75%) |  |  |
| Any decrease | 16 | 5 (31.2%) | 1.58 (0.29, 10.04) | 0.605 | 11 (68.8%) | 1.48 (0.30, 7.46) | 0.631 | 15 (93.8%) | 1.15 (0.49, 2.67) | 0.748 |
| Any increase | 28 | 2 (7.1%) | 0.27 (0.03, 2.00) | 0.203 | 23 (82.1%) | 3.02 (0.64, 14.79) | 0.160 | 25 (89.3%) | 1.31 (0.58, 2.97) | 0.522 |

**Supplementary Table S6.** Logistic and Cox regression results for the association of changes in ^mut^KRAS ctDNA from baseline with objective response at week 8, death within 1 year and OS

|  | | Objective Response | | | Death in 1 year | | | Overall survival | | |
| --- | --- | --- | --- | --- | --- | --- | --- | --- | --- | --- |
| Variable | N | ORR (%) | OR (95% CI) | P | Numbers (%) | OR (95% CI) | P | Numbers (%) | HR (95% CI) | P |
| **^mut^*KRAS* ctDNA absolute change at day 8** | | | | | | | | | | |
| Any absolute increase | 8 | 1 (16.7%) |  |  | 7 (87.5%) |  |  | 8 (100%) |  |  |
| Any absolute decrease | 9 | 4 (50%) | 5.00 (0.47, 123.07) | 0.220 | 7 (77.8%) | 0.75 (0.02, 15.10) | 0.851 | 9 (100%) | 1.76 (0.58, 5.38) | 0.321 |
| **^mut^*KRAS* ctDNA absolute change at day 15** | | | | | | | | | | |
| Any absolute increase | 6 | 0 (0%) |  |  | 5 (83.3%) |  |  | 6 (100%) |  |  |
| Any absolute decrease | 9 | 2 (25%) | - | 0.997 | 7 (77.8%) | 0.75 (0.02, 15.10) | 0.851 | 9 (100%) | 1.36 (0.42, 4.34) | 0.607 |
| **^mut^*KRAS* ctDNA absolute change at day 29** | | | | | | | | | | |
| Any absolute increase | 1 | 0 (0%) |  |  | 0 (0%) |  |  | 1 (100%) |  |  |
| Any absolute decrease | 17 | 5 (31.2%) | - | 0.997 | 15 (88.2%) | - | 0.999 | 17 (100%) | 1.68 (0.20, 13.95) | 0.632 |
| **^mut^*KRAS* ctDNA detection change at day 8** | | | | | | | | | | |
| No change | 16 | 5 (38.5%) |  |  | 13 (81.2%) |  |  | 16 (100%) |  |  |
| Detectable to not detectable | 1 | 0 (0%) | - |  | 1 (100%) | - |  | 1 (100%) | - |  |
| **^mut^*KRAS* ctDNA detection change at day 15** | | | | | | | | | | |
| No change | 11 | 1 (11.1%) |  |  | 9 (81.8%) |  |  | 11 (100%) |  |  |
| Detectable to not detectable | 3 | 1 (33.3%) | - |  | 3 (100%) | - |  | 3 (100%) | 1.89 (0.42, 8.38) | 0.404 |
| Non-detectable to detectable | 1 | 0 (0%) | - |  | 0 (0%) | - |  | 1 (100%) | 0.70 (0.08, 6.06) | 0.742 |
| **^mut^*KRAS* ctDNA detection change at day 29** | | | | | | | | | | |
| No change | 10 | 2 (22.2%) |  |  | 7 (70%) |  |  | 10 (100%) |  |  |
| Detectable to not detectable | 8 | 3 (37.5%) | 2.54 (0.25, 34.32) | 0.439 | 8 (100%) | - |  | 8 (100%) | 1.91 (0.58, 6.35) | 0.290 |

**Supplementary Table S7.** Summary of longitudinal analysis of ^mut^KRAS ctDNA with OS using joint model

|  | Model 1 |  |  | Model 2 |  |
| --- | --- | --- | --- | --- | --- |
| **Terms** | **Estimate** | **p-value** |  | **Estimate** | **p-value** |
| **Longitudinal sub-model** |  |  |  |  |  |
| Timepoint | -0.072 (0.01) | < 0.001 |  | -0.071 (0.01) | < 0.001 |
| Timepoint:armABX/GEM Concomitant | 0.014 (0.01) | 0.298 |  | 0.011 (0.01) | 0.407 |
| **Survival sub-model** |  |  |  |  |  |
| Concomitant (vs seq) | 0.28 (0.46) | 0.540 |  | 0.69 (0.33) | 0.034 |
| ^mut^*KRAS* ctDNA | 0.024 (0.01) | 0.057 |  | -0.012 (0.01) | 0.337 |
| ^mut^*KRAS* ctDNA detected | 1.754 (0.42) | < 0.001 |  |  |  |

Note: difference between model 1 and model2 is that the former adjusted baseline ctDNA status in the survival sub-model.
